# Wildland fire smoke alters the composition, diversity, and potential atmospheric function of microbial life in the aerobiome

**DOI:** 10.1038/s43705-022-00089-5

**Published:** 2022-01-25

**Authors:** Leda N. Kobziar, David Vuono, Rachel Moore, Brent C. Christner, Timothy Dean, Doris Betancourt, Adam C. Watts, Johanna Aurell, Brian Gullett

**Affiliations:** 1 Department of Natural Resources and Society, University of Idaho, Coeur d’Alene, ID 83814 USA; 2 Department of Civil and Environmental Engineering, Colorado School of Mines, Golden, CO 80401 USA; 3 Department of Microbiology and Cell Science, University of Florida, Gainesville, FL 32611 USA; 4 U. S. Environmental Protection Agency, Office of Research and Development, Research Triangle Park, NC 27711 USA; 5 Pacific Wildland Fire Sciences Laboratory, USDA Forest Service, Seattle, WA 98103 USA; 6 University of Dayton Research Institute, 300 College Park, Dayton, OH 45469 USA; 7 Present Address: Earth and Atmospheric Sciences, Georgia Institute of Technology, Atlanta, GA 30332 USA

**Keywords:** Air microbiology, Microbial ecology

## Abstract

The atmosphere contains a diverse reservoir of microbes but the sources and factors contributing to microbial aerosol variability are not well constrained. To advance understanding of microbial emissions in wildfire smoke, we used unmanned aircraft systems to analyze the aerosols above high-intensity forest fires in the western United States. Our results show that samples of the smoke contained ~four-fold higher concentrations of cells (1.02 ± 0.26 × 10^5^ m^−3^) compared to background air, with 78% of microbes in smoke inferred to be viable. Fivefold higher taxon richness and ~threefold enrichment of ice nucleating particle concentrations in smoke implies that wildfires are an important source of diverse bacteria and fungi as well as meteorologically relevant aerosols. We estimate that such fires emit 3.71 × 10^14^ microbial cells ha^−1^ under typical wildfire conditions in western US forests and demonstrate that wildland biomass combustion has a large-scale influence on the local atmospheric microbial assemblages. Given the long-range transport of wildfire smoke emissions, these results expand the concept of a wildfire’s perimeter of biological impact and have implications to biogeography, gene flow, the dispersal of plant, animal, and human pathogens, and meteorology.

## Introduction

Wildland fires (wildfires and prescribed fires) have been investigated for their terrestrial biophysical and atmospheric physiochemical impacts, but recent research that shows they are sources of bioaerosols (i.e., microbes and biogenic particulates) implies that fire may play roles in biological dissemination, microbial dispersal, and atmospheric processes [1–3]. The potential role of smoke as a vector to transport viable microbes challenges the concept that the immediate ecological effects of a wildland fire are predominantly confined to its perimeter [2–5]. Studies of low-intensity prescribed fires indicate that viable microbes emitted in smoke from biomass burning are both quantitatively and qualitatively different from the bioaerosols observed in ambient, smoke-free air [2, 6], implying that wildland fire may be a previously unrecognized mechanism for biological dispersal. In addition to the implications for dissemination, the meteorological roles of certain bioaerosols in the atmosphere, such as ice nucleating particles (INPs), may be accelerated by the aerosolization mechanisms of wildland fire [6]. Since high-intensity fires can loft smoke into the free troposphere [7] and across continents [8], the effects of this phenomenon are unlikely to be restricted to local scales. A better understanding of the microbes transported in smoke is relevant to diverse disciplines investigating microbial dispersal and its role in community ecology, pathogen spread and epidemiology [9–11], and the meteorological effects of fire aerosols [12].

The high cell viability reported for microbes in smoke from low-intensity prescribed fires [6] may not be representative of higher severity fires, since viability is likely affected by a combination of factors including fire behavior (e.g., combustion stage and efficiency, intensity, fuels consumed [2]), atmospheric conditions (e.g., RH, temperature, particulate matter [3]) and the tolerance of the aerosolized microbes to environmental stress (e.g., desiccation and heat [13, 14]). Indicators of increased microbial tracers (e.g., sucralose and mannitol) during biomass-burning seasons vs. non-smoke periods have also been found in air pollution studies around the globe [15, 16]. Further, recent work showing that ice nucleation particle (INP) concentrations in near-ground, wildland fire emissions are fivefold higher than those in ambient air suggests that INPs emitted in smoke plumes may be relevant drivers of ice phase precipitation [17]. While studies of wildland fire have demonstrated that incomplete combustion, convective wind generation, and/or advection of combustion products emit viable bioaerosols, the composition of microbes that wildland fire contributes to atmospheric biodiversity and its potential meteorological implications are unknown.

As part of the Fire and Smoke Model Evaluation Experiment (FASMEE) to fully characterize fuels, fire behavior, and emissions relationships [18, 19], we sampled and collected data directly over three high-intensity prescribed burns from 18 to 20 June 2019. The purpose of this study was to characterize the abundance, composition, viability, and ice nucleation activity of bioaerosols emitted in smoke plumes. Using a customized aerosol sampling payload [20] flown aboard an unmanned aircraft system (UAS), we sampled air before the fire (i.e., ambient air) and smoke (22 flights over three days) above a crown fire in a sub-alpine fir/aspen forest system in central Utah, USA. We used culture-independent approaches to examine the composition and structure of the microbial assemblages in smoke to answer the following questions: (1) How does smoke contribute to the diversity of atmospheric microbial aerosols? (2) What are the unique characteristics of the microbial assemblages emitted during high-intensity forest fires? (3) Does smoke contain certain groups of microbes that correlate to the INP concentration? (4) For the taxonomic groups enriched in smoke, does fire serve as a distinctive emission mechanism for these populations? The increased number and richness of microbes we document in smoke demonstrate a hitherto overlooked source of specific bioaerosols and suggest that wildfires are important spatiotemporal drivers of atmospheric biodiversity and meteorologically relevant aerosols.

## Materials and methods

### Site description

The prescribed fires were conducted by USDA Forest Service personnel in the Fishlake National Forest in Utah, USA (38° 25.224′, −112° 1.331′) between June 18 and 20th of 2019. The prescribed fire objective was to regenerate quaking aspen forest stands (*Populus tremuloides*) post-burn. Vegetation in the forests burned was dominated by sub-alpine fir (*Abies lasiocarpa*) and standing insect-killed Engelmann’s spruce (*Picea Engelmannii*) with patches of quaking aspen and sparse understories comprised of various shrubs and grasses. The area had not been burned for over 50 years and was suffering from an outbreak of western spruce budworm; both conditions led to a high buildup of large downed woody fuels which contributed to the intensity of the burns. Ignitions included combinations of ground-based “terra torches” and helicopter aerial ignitions. Ignited areas were allowed to burn together driven by wind and topography alignment to maximize fire intensity and consumption.

### Aerosol sampling

A Matrice 600P (DJI Industries) UAS was used to access smoke plumes during the sampling period, as well as the day prior for ambient air samples. On June 18th, eight non-smoke aerosol samples were taken at randomized locations within or above the burn units, including three samples taken at 3 m height above ground level (a.g.l.) under the tree canopy and five samples taken between 35–50 m AGL. During June 19th two units sized 13 and 20 ha were burned and 14 flights were conducted to sample smoke aerosols. On June 20th, the 982 ha Manning Creek Unit was burned and three flights were conducted. Sampling duration was limited to 10–15 min per flight due to operational considerations (sharing airspace with manned fire control aircraft). Fuel composition and load did not differ among locations, which were less than ½ mile distanced. All ignitions targeted the development of high-intensity crown fire behavior, with flame lengths ranging from 5–50 m.

Airborne measurements of partitioned particulate matter (PM; PM_1_, PM_2.5_, and PM_10_), RH, and air temperature were conducted using a customized payload designed for UAS applications [20]. Data were collected onboard the UAS and telemetered to a ground-based control unit; the payload also incorporates a GPS unit so that data can be time-synchronized with other measurements. Particulate matter values were corrected to better estimate values that might be obtained from a federal reference method using an average of reported correction factors from Sayahi et al. for the Plantower PMS 5003 [21]. All particulate matter data used in this study were for comparative analyses and do not intend to represent 24-h PM values.

A Leland Legacy compensating programmable vacuum pump attached to the Button Personal Aerosol Sampler (both from SKC, Inc., Eighty-Four, PA, USA) were attached to the undercarriage of the UAS [3] and used to sample at the prescribed volumetric rate of 4 L/min for aerosols <100 µm onto sterile 1 µm, 25 mm polycarbonate filters. Flow rates were calibrated prior to sampling [19]. Before each flight, all sampling encasings were sanitized with 80% ethanol and sealed in aseptic plastic bags. After each flight, the smoke-exposed filters were extracted from the samplers in a portable aseptic glovebox and stored in sterile cryovials in coolers for transport to the laboratory. For each flight, the Button samplers were cleaned with 80% ethanol inside the glovebox and allowed to thoroughly dry, and unused, sterile polycarbonate filters were inserted using aseptic techniques. The filter housing was then covered with a clean cap (not touching the filter) until installed on the UAS immediately prior to flight. Pumping was delayed until the UAS was in the target air type (smoke, ambient). Of two Buttons on each flight, one filter was used for molecular analyses, while the other was used for cell counts, viability assessments, and estimates of INPs. Three procedural blanks were collected using the same methods used for the smoke and ambient samples. To collect the blanks, the sampling devices were first cleaned with 80% ethanol as they would have been prior to loading new filters for the air samples. Once dry, new filters were installed and closed in the sampling devices and allowed to sit in the sampler for 3 min. They were then removed from the sampling devices and deposited in vials using the same procedures as used for the air samples in a portable glovebox. All blanks and air samples were stored, amplified, and sequenced identically. Because the smoke microbiome has not been characterized in the literature, we took a conservative approach to potential contamination and removed all taxa found on blanks from our analysis. A list of taxa removed can be found in Table S1. A total of 38 ASVs were removed from analyses.

### Cell counts and ice nucleation particles

To determine microbial and INP abundance, one of the filters from each UAS flight was suspended in clarified (passed through 0.2 µm syringe filter) phosphate-buffered saline. Half of the sample was then filtered in triplicate onto black 0.22 µm polycarbonate filters. The other half of the sample was aliquoted into 50 ml droplets and placed into the wells of a 96-well plate. The cells collected on the filters were fluorescently stained with SYBR Gold [6]. The filters were affixed to glass microscope slides and visualized using an epifluorescence microscope. Sixty random (computer-generated) fields of view were enumerated per filter and used to estimate the total number of cells per sample [6]. The number of cells per sample and the initial sample volume were used to calculate the cell concentration per cubic meter of air for cells <100 µm (cut-off diameter for SKC Button sampler). Blanks collected in the field were used to assess the number of potential contaminant cells: these were subtracted from all totals (360 ± 54 cells per filter). Immersion freezing assays were conducted with the plated samples to estimate the total concentration of INPs >1 µm that induce freezing at temperatures from −4 to −15 C. The plate was first sealed with a clear adhesive film and placed in a refrigerated ethylene-glycol bath. Measurements for each sample were taken in triplicate (technical replicates). To indirectly estimate the number of biological INPs, we conducted immersion freezing assays on identical samples after incubating the samples at 100 C for 15 min, a method used in prior studies to differentially reduce proteinaceous or heat-sensitive INPs without affecting mineral INPs [6]. The difference between the heated and non-heated results was inferred to be attributable to biological INPs.

### Smoke modeling

Following methods of Moore et al. [6], we used the First Order Fire Effects Model (FOFEM v. 6.7 [22]) to project the number of cells aerosolized by wildland fire burning these forests under 90th percentile weather and fuels conditions typical of wildfire occurrences in the region (determined using RAWS weather station for fire season).

### Cell viability

Cell viability was assessed through culture-independent methods using a live/dead stain. Briefly, cells were stained on black 0.22 µm polycarbonate Isopore filters using equal volumes of SYTO 9 and propidium iodide. Stained cells were then viewed using a Nikon ECLIPSE Ni epifluorescence microscope in sixty random fields of view. Cells that stained fluorescently green (and not red) were considered intact and viewed cells were inferred to be viable. All cells (viable or not) were quantified in relation to the total volume of air sampled.

### DNA extraction, amplification, and sequencing

Genomic DNA was extracted from 1 µm polycarbonate bioaerosol filters (one from each flight, from the coupled two-filter sampling array) using the ZymoBIOMICS DNA/RNA Miniprep kit (Zymo Research Corp, Irvine, CA) following the manufacturer’s protocol. Extraction blanks were used as a negative controls and ZymoBIOMICS Microbial Community Standard were used as positive controls. The ribosomal ITS region and V3-4 region of small-subunit rRNA gene (16S and 18S) were used to identify the fungal, bacteria/archaea, and eukaryotic organisms, respectively, using the ZymoBiomics Targeted Sequencing Service. Briefly, PCR reactions were performed on real-time PCR machines to control cycles and prevent PCR chimera formation. The final PCR products were quantified with qPCR fluorescence readings and pooled together based on equal molarity. The final pooled library was cleaned with the Select-a-Size DNA Clean & Concentrator™ (Zymo Research, Irvine, CA), then quantified with TapeStation^®^ (Agilent Technologies, Santa Clara, CA) and Qubit^®^ (Thermo Fisher Scientific, Waltham, WA). Following library preparation, sequencing was performed on the Illumina^®^ MiSeq™ with a v3 reagent kit (600 cycles) with >10% PhiX spike-in.

### Bioinformatics analysis

Amplicons generated from Illumina sequencing were processed in the DADA2 pipeline [23] using the full amplicon workflow under default conditions: quality filtering, dereplication, sample inference, chimera identification/ removal, and merging paired-end reads. Taxonomic assignments were accomplished with Uclust in QIIME v.1.9.1 and multiple sequence alignment was performed using the Zymo Research Database and reference alignment, respectively. Taxa found in procedural blanks were filtered from all samples in the taxa table. Data analysis and visualization were performed in the R environment for statistical computing using microbiome-specific packages (phyloseq, microbiome, ampvis2, and vegan), as well as R custom scripts. Sequence read statistics are shown in Table S2, while sequencing depth for each sample was verified using rarefaction (Fig. S1).

### Statistical analyses

Analyses were conducted in the R environment and with NCSS 2021 Statistical software. Differences between live/dead, total cells, read abundances, PM, environmental conditions, and INPs between air types (ambient vs. smoke) were tested for normality using the Kolmogorov–Smirov and Shapiro–Wilk tests. In cases where the null hypothesis was rejected, suggesting the data came from a non-normal distribution, non-parametric statistical tests were used and Welch’s ANOVA was used where variances differed between test groups. Relationships were assessed using Pearson’s correlation coefficients. Significance was at the *p* < 0.05 level unless otherwise noted.

### Data sharing

Sequence data generated from this work is deposited and available in the NCBI database under the BioProject number PRJNA793272.

## Results and discussion

### Fire conditions and particulate and bioaerosol emissions

**Fig. 1:**
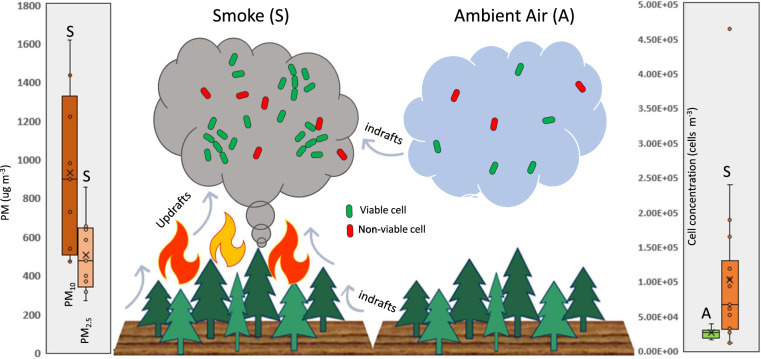
Particulates and microbial cells in smoke and ambient air across three days of high-intensity forest fire in Utah, USA. Drawing shows scaled concentrations of cells contrasting ambient air (“A”, mean sampling height above ground level 25 m, *N* = 8) and smoke plumes (“S”, mean height 75 m, *N* = 17). Number of viable and non-viable cells differed significantly between air types (K–S test; *P* < 0.01). Particulate matter concentrations (µg m^−3^) in smoke were nearly three orders of magnitude higher (K–S test; *P* < 0.005) than in ambient conditions for PM_10_ and significantly higher for PM_2.5_ and PM_1.0_ size fractions (data not shown).

Fire radiative power values estimated from satellite imagery ranged from 6 to 259 MW over three days of burning [19]. Smoke sampled above combusting vegetation contained high concentrations of PM_10_ (mean ± s.e. 928.4 ± 140.6 µg m^−3^; Fig. 1). Microbial cells are a component of total bioaerosols, and their abundance can correlate with PM in ambient conditions [24] as well as in wildland fire smoke [6]. However, we observed that only the concentration of viable cells (and not total cells) correlated with PM_2.5_ and PM_10_ values (*r*^2^ = 0.80, and 0.81, respectively; *p* < 0.03) in all air samples, while PM_2.5_ and PM_10_ were weakly correlated with viable cell abundance in smoke (*p* < 0.10). If cell aggregates are attached to individual particles as reported previously [6], it might be inherently difficult to detect linear correlations between these aerosol types.

Temperature, relative humidity (RH), and wind speed are conditions that influence microbial aerosol emissions, as well as their composition, abundance, and viability [25]. In general, temperature, wind speed, and RH are higher in smoke plumes compared to surrounding air masses [26]. During our flights, environmental conditions in-plume generally followed these expectations. However, none of the environmental variables measured could explain the variation in cell concentrations observed within either air type. Ground-level (2 m) wind speeds pre-burn and during smoke sampling flights did not differ significantly, (2.5 ± 0.1 m s^−1^ vs. 4.0 ± 0.2 m s^−1^, *p* < 0.05), suggesting any confounding effects of background (i.e. not fire-generated) winds on microbial aerosolization patterns were similar between ambient and smoke samples. It is likely that cell concentration variability was driven by a complex interaction of environmental conditions, fire behavior, and which fuels were undergoing active combustion at the time of sampling.

The concentrations of cells presented in Fig. 1 are based on the total number of cells observed per volume of air sampled minus the number of cells observed in procedural blanks. The cell concentration in smoke sampled during flights (mean ± standard error; 1.02 ± 0.26 × 10^5^ m^−3^) was significantly higher (Mann–Whitney *U* test; *p* < 0.02) than in air sampled prior to burning (2.6 ± 0.3 × 10^4^ m^−3^). The high coefficient of variation for the smoke cell data (25%) is similar to that of previous studies [6] and likely reflects the dynamic nature of fire behavior. For example, crown fire fuel types support both torching and intermittent crown fire that create highly variable and turbulent winds [27]. The cell concentrations observed in smoke are about twice those reported from ground-based samples of small, low-intensity prescribed fire smoke in grass/shrub fuels in Florida, USA [6]. The higher values in this study imply that differences in fuel composition/consumption coupled with a higher fire intensity and area burned produce higher cell concentrations, as is also observed for wildfire dust emissions [28]. In contrast to <5 Mg ha^−1^ fuel consumption values typical of prescribed burns in the Florida fuel type [29], fuel consumption values determined in this study using aerial LiDAR averaged 59.7 Mg ha^−1^, with a 90th percentile value of 127.2 Mg ha^−1^ (Hudak & McCarley, personal comm.). Higher cell and particle concentrations may also be due to collecting aerosol data and samples directly above and from within smoke plumes, rather than downwind using ground-based sampling locations that were ~30 m outside the combustion zone in the Florida study [6]. Based on smoke modeling projections (FOFEM v 6.7: [22]) under typical 90% wildfire weather conditions, we estimate that 3.71 × 10^14^ cells ha^−1^ were aerosolized, which is fivefold higher than values estimated for the prescribed burns in Florida [6].

Based on epifluorescence microscopy of samples stained with SYTO 9 and propidium iodide to distinguish total and dead cells, respectively, cell viabilities of 69 ± 17% were inferred for ambient air, whereas 78 ± 5% of cells in smoke appeared to be viable (Fig. 1). There were 3.4 times as many viable cells in smoke versus ambient air (6.2 ± 1.3 × 10^4^ m^−3^ and 1.8 ± 0.434 ×10^4^ m^−3^, respectively). Cell concentrations were not significantly different across sampling altitudes of 40 to 150 m above the combustion zone, indicating that microbial aerosols within the smoke plumes sampled were relatively well mixed. Although the abundance of microbial cells in smoke was higher than those reported in the afore-mentioned Florida burns, the fraction of cells inferred to be viable (Fig. 1) was similar (80%; (5)). Given the high intensity of the fires sampled and the sensitivity of many microorganisms to high temperatures, this result is unexpected. Although temperatures in crown fires can exceed 1000 °C [30], there is high variability of temperature at sub-meter scales [31, 32]. At scales applicable to particulate aerosols, microorganisms may be protected from high exposure to heat and water loss by the materials they are attached to (e.g. particles of mineral soil or plant tissue). Alternatively, the microbes found in smoke may be advected from soils and plant surfaces from outside the combustion zone and mixed with incompletely combusted particulate matter in the smoke plume as a result of “pyro-convection” [28].

Vertical air mixing due to combustion aerosolizes micro- to macroscopic particles [33] from plant material as well as organic and mineral components of soil [28, 34]. For example, the smoke plume of the high-intensity Biscuit Fire in Oregon, USA, was estimated to aerosolize 127 Mg·ha^−1^ of fine mineral soil and transport it across the Pacific Ocean [34]. Field measurements indicate that as a fire front progresses, combustion increases air buoyancy that induces strong vertical lifting in smoke columns followed by a weaker subsidence forming behind the fire front [29]. A plume from the El Portal wildfire in Yosemite National Park, CA induced updraft winds of 13.5 m s^−1^, and horizontal indraft into the convective column extended over a kilometer from the center of the plume [35]. Since wind speeds as low as 2–3 m s^−1^ are sufficient to aerosolize microbial cells from soils or plant surfaces [25], higher values associated with intense fires should significantly increase the contribution of bioaerosols sourced both external and internal to the region of combustion.

### Microbial diversity of wildland fire smoke

#### Bacterial and archaeal assemblages

**Fig. 2:**
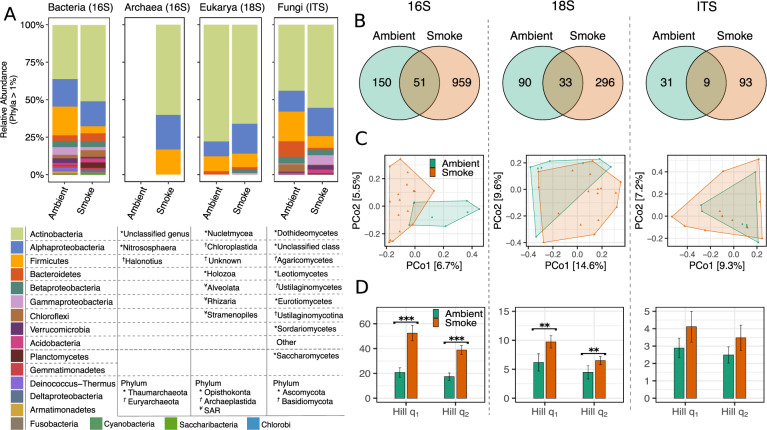
Species richness, diversity and taxa composition of microbial cells characterizing smoke (*N* = 17) and ambient air (*N* = 8) conditions. Taxa composition between ambient air and smoke (**A**). Venn diagrams for total, unique, and shared phylotypes between ambient air and smoke Samples for each sequencing region (**B**). PCoA of community similarity between ambient and smoke (**C**). Hill diversity comparison between ambient and smoke (error bars represent the standard error) (**D**).

**Fig. 3:**
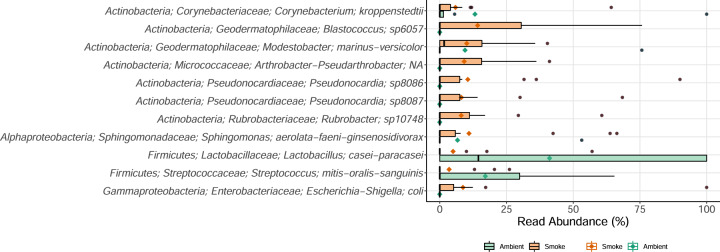
Abundance of the 11 most abundant species-level ASVs, belonging to either ambient (*N* = 8) or smoke (*N* = 17) libraries, that are detected in at least 20% of the samples. Solid bars within boxplot represent the median read abundance and colored diamonds represent the mean read abundance of each air type.

The phylum-level taxonomic composition of the bacteria in smoke and ambient air was dominated by members of the Actinobacteria, which comprised 50.7 ± 2.9% and 36.1 ± 4.8%, respectively, of the total sequences (Fig. 2A; SI Appendix Figs. S2, S3). Bacteroidetes (5.3 ± 1%), Chloroflexi (4.4 ± 1%), Planctomycetes (3.6 ± 0.9%), Acidobacteria (2.6 ± 0.8%), and Deltaproteobacteria (1.3 ± 0.7%) were all more abundant in smoke than in ambient air (Fig. 2A, SI Appendix Fig. S2). Archaea were only recovered in smoke, with Thaumarchaeota representing 0.4 ± 0.2% of the read abundance. Firmicutes were significantly higher in ambient air (19.1 ± 4.2%) than in smoke (4.7 ± 0.8%). However, despite having overall lower abundance in smoke, there was higher diversity of distinct Firmicute families associated with the spore-forming orders *Bacillales* and *Clostridiales* in smoke [36] (Fig. S2). Elevated proportions of Actinobacteria, Firmicutes, and Proteobacteria have also been reported in other aerobiology studies, such as in clouds [37], intercontinental dust [38], and in rural versus urban centers [39, 40]. In our study, Actinobacteria were significantly enriched in smoke above background levels. This could be due to morphological features that facilitate aerosolization, such as the production of mycelial hyphae and spores in many known strains of Actinobacteria [41]. Furthermore, smoke was enriched with bacteria phylogenetically related to taxa with known associations to soils and the phyllosphere [6], such as ammonium oxidizing archaea within the Thaumarchaeota (Fig. 2A), Actinobacteria families *Geodermatophilaceae, Kineosporiaceae, Intrasporangiaceae, Solirubrobacteraceae*, and *Pseudonocardaceae*, and Alpha- and Betaproteobacteria families *Sphingomonadaceae*, *Bradyrhizobiaceae*, and *Nitrosomonadaceae* (Figs. 2A, 3, SI Appendix Figs. S4, S5). This observation is consistent with these bacteria being sourced from soil and plants within the local species pool, as suggested by Bowers et al. for non-smoke bioaerosols [40]. In ambient air, *Lactobacillus sp*. and *Streptococcus sp*. were found in higher read abundance over smoke samples (Fig. 3). *Lactobacillus* is commonly associated with fermentation products but along with many *Streptococci* is also associated with oral microbiota. However, numerous ASVs of *Lactobacillus* and *Streptoccocus* genera were also identified in environmental sources (soil, plants) characterized by Dove et al. in locations neighboring our study units [42], highlighting the relative ease by which these organisms may be transported in air.

A higher proportion of the bacterial phylotypes were unique to smoke than to ambient air (959 versus 150, respectively), with only a small fraction shared between the air types (Fig. 2B). Bacterial libraries also differed significantly between ambient and smoke with regards to community structure (*r*^*2*^_ADONIS_ = 0.06, *p* = 0.001; Fig. 2C) and diversity metrics (*t*-test, Hill-q_1_: *p* < 0.001, Hill-q_2_: *p* < 0.001; Fig. 2D), with Actinobacteria, Alphaproteobacteria, Firmicutes, and Bacteroidetes driving most of the inter-sample variation (SI Appendix Fig. S3). Together with observations that wildland fires aerosolize viable microbes (Fig. 1), the molecular results show that assemblages of bacteria and archaea in smoke are more diverse (Fig. 2) and distinct from those found in ambient air. Model simulations of airborne microbial dispersal suggests that intra-hemispheric transport of particles 10-20 µm in diameter can be successfully distributed over one year [43] and if shielded by inclusions within particulate matter, may be able to survive UV irradiation in the upper atmosphere [44]. As smoke contained numerous phylotypes closely related to potential human or plant pathogens, including *Bacillus anthracis-cereus, Pseudomonas syringae, Streptococcus spp., Escherichia-Shigella coli, Corynebacterium jeikeium, Acinetobacter ursingii, Haemophilus haemolyticus-influenzae*, and some *Staphylococcus spp*. (the last genus also appeared in ambient air), long-distance and long-duration transport of fire-vectored microbes may have implications for global health.

#### Eukaryotic assemblages

The structure and diversity of eukaryotic assemblages based on 18S rRNA gene sequence analysis differed between ambient and smoke samples (*r*^*2*^_ADONIS_ = 0.07, *p* = 0.01; Fig. 2B; *t*-test, Hill-q_1_: *p* = 0.04, Hill-q_2_: *p* = 0.08; Fig. 2C, D) although at a weaker significance level than observed for bacterial assemblages. The eukaryotic libraries were dominated by fungal taxa (78 ± 8.4 and 66 ± 4.2% Nucletmycea for ambient air and smoke, respectively; Fig. 2A, Fig. S6A), and differences in composition between the ambient and smoke samples was primarily due to the abundance of sequencing reads from the plant phylum Chloroplastida (9.9 ± 5.1% and 19.9 ± 3.9%, respectively; Fig. 2A, Fig. S6A). The relative abundance of Chloroplastida phylotypes in smoke did not correlate with the viable or total cell concentrations, with 61 sequences (74%) being closely related to Pinales. Sequences in the Pinales order comprised over 10.8% (75,851 reads) of the total 18S reads in smoke, compared to only 2.7% (6336 reads) in the ambient air. As Pinales includes a majority of the dominant tree species burned in the site (e.g., *Abies, Picea* spp.), this difference indicates that tree components (e.g., partially combusted litter, downed woody debris, live and dead standing trees, and pollen) associated with wildland fire were a direct source of bioaerosols in smoke. The low abundance of Pinales in ambient air (found only in one ambient sample vs. 11/17 samples in smoke) may indicate pollen serving as the source of genetic material collected under background conditions, as would be consistent for the season and location [45] and considering the unlikely potential for the low ambient wind speeds observed to aerosolize other plant materials during ambient air sampling. There is a critical need for approaches that accurately track specific combustion sources in smoke, and chemical speciation of aerosol-phase emissions has proved useful for this purpose [46], but the extent to which aerosol chemistry alone can be used for apportionment of biomass-burning constituents is limited. The results of this study indicate that genetic material from fuel sources remains sufficiently preserved in smoke for DNA sequence characterization, demonstrating the potential for a novel alternative for tracking the contribution of specific wildland fires (with known fuel types) to air pollution.

ITS-based characterization of fungal taxa showed that Ascomycota made up 68.7 ± 12.4% and 80.9 ± 6.4% of ambient and smoke libraries, respectively, and their prevalence over Basidiomycota has been reported in some [47, 48] but not all aerobiological studies [49, 50]. Basidiomycota were more abundant in ambient air (29.2 ± 12.7%) than in smoke (12.5 ± 3.9%) (Fig. S6B). ITS sequencing identified a total of 133 fungal taxa across all samples: 93 of these were only found in smoke, 31 taxa were unique to ambient air, and 9 taxa were shared between smoke and ambient samples (Fig. 2B). Although volume of air sampled here is significantly lower than in non-smoke aerobiology studies, these estimates of richness are only moderately lower than those reported in other aerobiology investigations based on aerosols derived from larger air volumes and longer sampling durations (e.g. >1000 L) [49]. For example, Frohlich-Nowoisky et al. [49, 51] reported 368 species resulting from filtering 3 M L of air in a single location in Europe four times over a year. The approximately fivefold difference in number of cells in smoke versus ambient air samples in this study likely explains the relatively high number of taxa found even in the low volumes of smoke.

Similar to previous culture-independent studies of fungal heterogeneity in non-smoke air, most (95%) of the phylotypes detected were found in only one sample (e.g. compared to 70% in previous work [49]). This suggests that either the actual richness of fungi in smoke was underestimated by the sampling strategies used in this study, or that the dynamic nature of fuels consumption (oscillating between surface and crown fuels) emits non-overlapping source assemblages of organisms as fire moves across a landscape. There were are no detectable patterns in the phylotypes aerosolized in relation to time of day or among samples collected synoptically. Moreover, as reported in a previous study of fungi in settled dust from across the USA [48], a significant portion of the fungal families was unclassified (42.8% in smoke, 39% in ambient air), suggesting considerable challenges to characterizing species richness in bioaerosol samples due to limited reference libraries [48]. This may help explain why the ITS libraries did not differ across air types in either community structure (*r*^*2*^_ADONIS_ = 0.04, *p* = 0.76; Fig. 2C) or diversity (Mann–Whitney *U* test, Hill-q_1_: *W* = 55.5, *p* = 0.2, Hill-q_2_: *W* = 57.5, *p* = 0.3; Fig. 2D).

The bulk of the Basidiomycota reads in smoke were members of the Agaricomycetes and Ustilaginomycetes classes (68% and 16%, respectively). Dothideomycetes comprised 55.5% of the total sequences in smoke with Eurotiomycetes making up 6.3% (Fig. S6B). Compared to a global analysis of the continental airborne fungi [50], smoke Basidiomycota were more evenly represented across multiple classes rather than being dominated by Agaricomycetes, which reportedly comprised 84% of Basidiomycota bioaerosols across multiple continents. The identities and relative composition of the fungal families aerosolized in smoke differed from those reported by Dove et al. [42] for a nearby (Utah, USA) microbiome and study of soil and phyllosphere microbiomes. Of the top ten classified families found in smoke in this study, Davidiellaceae, Dothioraceae, Malasseziaceae, Helotiales and Pezizomycotina were not among the top ten in the leaf, stem, or fine root samples examined in the same ecosystems at nearby locations. These results suggest that wildland fire emits fungal assemblages that differ from their source communities in species relative composition, as would be expected due to the temporal and spatial heterogeneity of fuels consumption as well as differences in heat/desiccation tolerance and aerosolization potential among taxa.

A total of 48 out of 101 classifiable genera were found in smoke compared to 27 out of 92 in ambient air. *Cladosporium* is a genus common in bioaerosols and was the most frequently observed genus shared between ambient and smoke air (found in 80% of samples), while *Aureobasidium* spp., *Alternaria* spp., and *Elasticomyces* spp. were the only genera found in three or more of the smoke samples. Read abundance in smoke was highest among *Auerobasidium*, *Ramularia*, and *Dothidea* genera. Potential allergens and plant or human pathogens found in smoke included *Cladosporium* spp., *Alternaria* spp., *Ustliago hordei*, *Aspergillus* spp., *Aspergillus penicillioides, Cladophialophora* spp., *Ochroconis* spp., and *Candida sake*; the latter four found only in smoke. If smoke aerosols containing viable cells of these microorganism are deposited within or beyond the burned area, post-fire dynamics may be impacted. Since *Cladosporium* and *Alternaria* include phytopathogenic species, sources that increase their abundance in the phyllosphere following high severity fire could have relevance to post-fire aspen recovery [42].

#### Variability of taxa between sample type and across domains

**Fig. 4:**
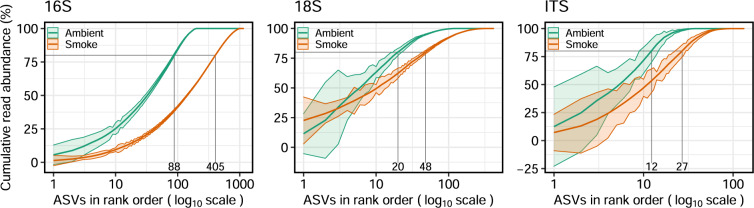
Relationship between rank order and cumulative read abundance of microbial taxa in smoke (*N* = 17) versus ambient (*N* = 8) air samples. Rank order (log10 scale) of taxa plotted against their mean cumulative read abundance for ambient and smoke for each target region: 16S, 18S, and ITS. Ribbons represent the standard deviation of taxa prevalence across sample type. The number of taxa representing 80% of the diversity within each sample type is shown.

To investigate the distribution and variability of abundant and rare taxa within each air type and domain, the rank order of each ASV was plotted against its cumulative read abundance (i.e., individual organisms within each ASV) (Fig. 4). This analysis enabled a direct comparison of variation within smoke and ambient air types (i.e., prevalence and consistency of taxa observed within air types), diversity between each air type, and of data obtained using different molecular approaches (i.e., for bacteria, fungal, and eukaryotic taxa). Based on the small-subunit rRNA and ITS regions analyzed, smoke samples were more diverse, particularly within ≥80% cumulative read abundance. Among the top ten most abundant taxa, we observed a high degree of variation within each air type, indicating that taxa were not uniformly found in consistent proportions, apart from bacterial and archaeal smoke taxa. The consistent prevalence (i.e., the likelihood of consistently sampling aerosols with the same proportion of taxa) of bacteria and archaea across smoke samples (see lower variation in Fig. 4) suggests these taxa may possess physiological or trait-specific fitness advantages amenable to aerosolization and survival during atmospheric transport. This might imply that a deeper and/or more abundant source pool of bacterial single cells, hyphae, or spores are aerosolized more easily from native sources in association with combustion processes leading to a more consistent and diverse microbial assemblage introduced to the atmosphere. Conversely, it appears that stochastic factors may be driving the distribution of eukaryotes and fungi in both ambient air and smoke, as well as bacteria in ambient air. Beyond aerosolization through combustion, the proximal air currents generated are likely to have less of a mitigating effect on the physical constraints associated with aerosolizing large particles [43]. The role of smoke in microbial dispersal and ecosystem function has not been previously considered, and given that the odds of colonization increase when immigrating microorganisms are abundantly sourced [52, 53], it represents new and fertile territory for future investigations [52]. Since climate change is increasing the risks, scale, and severity of wildfires in many regions, research addressing the dispersal of microorganisms by smoke and its ecological effects where deposited is timely.

### Ice nucleation particles and microbial composition

**Fig. 5:**
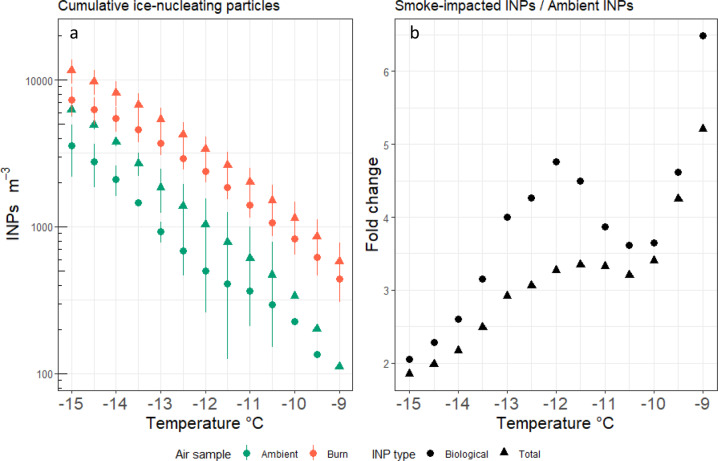
Ice nucleation activity of biological and non-biological particles in smoke and ambient air. Cumulative INPs m^-3^ in ambient air and smoke show higher percentage of INPs are biological in smoke and overall INPs are higher across all temperatures evaluated (**a**). Fold-change of INPs across different temperatures between smoke and ambient air trend towards increasing differences at higher temperatures (**b**).

The concentration of INPs was significantly higher in smoke (*n* = 17) than in ambient air (*n* = 4; *p* < 0.05), and smoke contained a larger proportion of INPs that were active across all temperatures > −16 °C (Fig. 5a). Fold-changes in INPs were highest at temperatures of −13 °C to −11 °C, as well as at −9 °C (Fig. 5b). In smoke and ambient air, total INPs were correlated with PM_1.0_ (Pearson’s *r* = 0.59; *p* < 0.05), but not to larger (PM_2.5_, PM_10_) particle size classes (*p* > 0.05). INPs were more abundant in warmer air masses with lower RH (*r*(21) = 0.62, *p* < 0.04 and r(21) = −0.64, *p* < 0.04, respectively), which corresponds to the conditions when smoke was sampled. The highest numbers of INPs corresponded to the sampling periods when observed fire behavior was most extreme (crown fire). The proportion of heat-sensitive INPs, inferred to be biological in origin [54] (labeled “Bio” in Fig. 5), was higher in smoke than in ambient air (Fig. 5; *p* < 0.05), similar to findings from low-intensity prescribed fire smoke [6] and higher altitude wildfire smoke plumes measured using manned aircraft [55]. Smoke from forest fires is a long-suspected source of INPs [12, 28]; however, only recently have data been available that show smoke-derived INPs are predominantly biological [6, 55].

Smoke samples with higher ITS phylotype richness correlated positively with the total (*r*(15) = 0.63, *p* = 0.009) and biological INP abundance (*r*(15) = 0.65, *p* = 0.007), while there was no correlation in samples of ambient air. Within the 18S assemblage, ASV read abundance of Chloroplastida and Nucletmycea taxa also correlated positively with total INPs (*r*(15) = 0.56 and 0.56, respectively, *p* < 0.02). Since cell concentration did not correlate with phylotype richness (*p* > 0.05) or number of reads (*p* > 0.10), this pattern in smoke is not easily explained by higher cell counts. Although the most well-known bacterial ice-nucleating species belong to the Gammaproteobacteria (e.g., species in the Pseudomonadaceae, Xanthomonadaceae, and Enterobacteriaceae families), this class was not at higher relative abundance in smoke samples and Pseudomonadaceae taxa were more abundant in the ambient air samples. Together, these results suggest that wildland fire emits atypical biogenic INPs that originate from diverse microbial sources.

## Conclusions

In this study, samples collected directly within smoke plumes above high-intensity forest fires were used to simultaneously characterize the airborne microbial assemblages and quantify INPs and particulate matter. We show that the processes associated with biomass fire are a source of unique and viable bioaerosols, adding to the growing body of evidence for wildland fire as a mechanism of microbial dispersal [2, 3, 6]. Under the extreme fire behavior conditions examined in this study, a diverse assemblage of viable microbes and INPs were emitted from the combustion environment and at significantly increased abundance than in the proximate atmosphere. These fires were clear sources of INPs but we did not see trends that implicate well-known bacterial INPs. The sources of these particles may be from bacteria and fungi, or even plant materials, not yet known to possess this phenotype. Investigations of smoke should continue to explore bioaerosol composition to identify new species that can serve as biological INPs (e.g. [56]).

Community and diversity differences in the bacteria, archaea, and fungi detected in ambient air versus smoke samples, as well as key differences in the relative abundance of certain fungal taxa, indicate that the combustion environment aerosolizes an assemblage that differs from that produced by other bioaerosol emissions processes. How microbes are aerosolized within and proximate to the combustion zone has not yet been studied but could reveal new insights about the traits and species relevant to observed patterns of dispersal, post-fire recovery, and the tolerance of specific organisms to the environmental stressors associated with the combustion environment (e.g., extreme heat, desiccation, and aerosolization).

Viewing wildland fire as a traceable environmental source of microbes to the atmosphere may assist with explaining local-to-global patterns of microbial distribution. Projections based on smoke plume transport models suggest that microbiota may be moved, along with other aerosols in the smoke, hundreds to thousands of miles from the combustion zone [57]. The deposition of these microorganisms, which we have shown are compositionally different than background bioaerosol composition, may have impacts on a wide array of sink biomes. Further studies that track microbes from source, through the atmosphere via smoke, and finally to their sinks would illuminate possible fire-mediated teleconnections among ecosystems and metacommunities. The microbial content of smoke includes a subset of taxa that could potentially be pathogenic to plants and humans, and the precise identity, deposition, and post-dispersal effects of these groups deserve further study.

## Supplementary information

Supplemental Material

## References

[1] Després Viviane R, Huffman JA, Burrows SM, Hoose C, Safatov Aleksandr S, Buryak G (2012). Primary biological aerosol particles in the atmosphere: a review. Tellus B Chem Phys Meteorol.

[2] Kobziar LN, Pingree MRA, Larson H, Dreaden TJ, Green S, Smith JA (2018). Pyroaerobiology: the aerosolization and transport of viable microbial life by wildland fire. Ecosphere.

[3] Kobziar LN, Pingree MRA, Watts AC, Nelson KN, Dreaden TJ, Ridout M. Accessing the life in smoke: a new application of unmanned aircraft systems (UAS) to sample wildland fire bioaerosol emissions and their environment. Fire. 2019;2:56.

[4] Mims SA, Mims FM (2004). Fungal spores are transported long distances in smoke from biomass fires. Atmosph Environ.

[5] McLauchlan KK, Higuera PE, Miesel J, Rogers BM, Schweitzer J, Shuman JK (2020). Fire as a fundamental ecological process: research advances and frontiers. J Ecol.

[6] Moore RA, Bomar C, Kobziar LN, Christner BC. Wildland fire as an atmospheric source of viable microbial aerosols and biological ice nucleating particles. ISME J. 2021;15:461–72. 10.1038/s41396-020-00788-8 PMC8027831 33009511

[7] Price OF, Purdam PJ, Williamson GJ, Bowman DMJS (2018). Comparing the height and area of wild and prescribed fire particle plumes in south-east Australia using weather radar. Int J Wildland Fire.

[8] Cottle P, Strawbridge K, McKendry I (2014). Long-range transport of Siberian wildfire smoke to British Columbia: lidar observations and air quality impacts. Atmosph Environ.

[9] Han X, Dendy SP, Garrett KA, Fang L, Smith MD (2008). Comparison of damage to native and exotic tallgrass prairie plants by natural enemies. Plant Ecol.

[10] Haselow DT, Safi H, Holcomb D, Smith N, Wagner KD, Bolden BB (2014). Histoplasmosis associated with a bamboo bonfire — Arkansas, October 2011. Morb Mortal Wkly Rep.

[11] Jeger MJ, Pautasso M, Holdenrieder O, Shaw MW (2007). Modelling disease spread and control in networks: implications for plant sciences. New Phytol.

[12] Petters MD, Parsons MT, Prenni AJ, DeMott PJ, Kreidenweis SM, Carrico CM, et al. Ice nuclei emissions from biomass burning. J Geophys Res Atmosph. 2009;114:D7.

[13] Brown GS, Mohr AJ. Fate and transport of microorganisms in air. In: Manual of environmental microbiology. Washington DC: ASM Press; 2016, p. 3.2.4-1–3.2.4-12.

[14] Pingree MRA, Kobziar LN (2019). The myth of the biological threshold: a review of biological responses to soil heating associated with wildland fire. For Ecol Manag.

[15] Yang Y, Chan C, Tao J, Lin M, Engling G, Zhang Z (2012). Observation of elevated fungal tracers due to biomass burning in the Sichuan Basin at Chengdu City, China. Sci Total Environ.

[16] Rajput P, Anjum MH, Gupta T (2017). One year record of bioaerosols and particles concentration in Indo-Gangetic Plain: Implications of biomass burning emissions to high-level of endotoxin exposure. Environ Pollut.

[17] Mülmenstädt J, Sourdeval O, Delanoë J, Quaas J (2015). Frequency of occurrence of rain from liquid-, mixed-, and ice-phase clouds derived from A-Train satellite retrievals. Geophys Res Lett.

[18] Prichard S, Larkin NS, Ottmar R, French NHF, Baker K, Brown T (2019). The Fire and Smoke Model Evaluation Experiment—A Plan for Integrated Large Fire–Atmosphere Field Campaigns. Atmosphere.

[19] Aurell J. Wildland fire emission sampling at Fishlake National Forest, Utah using an unmanned aircraft system. Atmosph Environ. 2021;247:118193. 10.1016/j.atmosenv.2021.118193 PMC8318188 34335074

[20] Nelson K, Boehmler J, Khlystov A, Moosmüller H, Samburova V, Bhattarai C (2019). A multipollutant smoke emissions sensing and sampling instrument package for unmanned aircraft systems: development and testing. Fire.

[21] Sayahi T, Butterfield A, Kelly KE (2019). Long-term field evaluation of the Plantower PMS low-cost particulate matter sensors. Environ Pollut.

[22] Reinhardt ED, Keane RE, Brown JK. First order fire effects model: FOFEM 4.0, user’s guide. Ogden, UT: U.S. Department of Agriculture, Forest Service, Intermountain Research Station; 1997.

[23] Callahan BJ, McMurdie PJ, Rosen MJ, Han AW, Johnson AJA, Holmes SP (2016). DADA2: high-resolution sample inference from Illumina amplicon data. Nat Methods.

[24] DeMott PJ, Prenni AJ, Liu X, Kreidenweis SM, Petters MD, Twohy CH (2010). Predicting global atmospheric ice nuclei distributions and their impacts on climate. PNAS.

[25] Jones AM, Harrison RM (2004). The effects of meteorological factors on atmospheric bioaerosol concentrations—a review. Sci Total Environ.

[26] Werth PA, Potter BE, Alexander ME, Clements CB, Cruz MG, Finney MA, et al. Synthesis of knowledge of extreme fire behavior: volume 2 for fire behavior specialists, researchers, and meteorologists. Portland, OR: U.S. Department of Agriculture, Forest Service, Pacific Northwest Research Station; 2016.

[27] Taylor SW, Wotton BM, Alexander ME, Dalrymple GN (2004). Variation in wind and crown fire behaviour in a northern jack pine – black spruce forest. Can J For Res.

[28] Wagner R, Jähn M, Schepanski K (2018). Wildfires as a source of airborne mineral dust – revisiting a conceptual model using large-eddy simulation (LES). Atmosph Chem Phys.

[29] Ottmar RD (2014). Wildland fire emissions, carbon, and climate: modeling fuel consumption. For Ecol Manag.

[30] Butler BW. Characterization of convective heating in full scale wildland fires. In: Viegas DX editor. (CD-ROM) Proceedings of the VI International Conference on Forest Fire Research; 15–18 November 2010; Coimbra, Portugal. Coimbra, Portugal: University of Coimbra; 2010.

[31] Sparks AM, Smith AMS, Talhelm AF, Kolden CA, Yedinak KM, Johnson DM (2017). Impacts of fire radiative flux on mature Pinus ponderosa growth and vulnerability to secondary mortality agents. Int J Wildland Fire.

[32] O’Brien JJ, Loudermilk EL, Hornsby B, Hudak AT, Bright BC, Dickinson MB (2016). High-resolution infrared thermography for capturing wildland fire behaviour: RxCADRE 2012. Int J Wildland Fire.

[33] Lynch JA, Clark JS, Stocks BJ (2004). Charcoal production, dispersal, and deposition from the Fort Providence experimental fire: interpreting fire regimes from charcoal records in boreal forests. Can J For Res.

[34] Bormann BTBT, Homann PSHS, Darbyshire RLDL, Morrissette BAMA (2008). Intense forest wildfire sharply reduces mineral soil C and N: the first direct evidence. Can J For Res.

[35] Lareau NP, Clements CB (2017). The mean and turbulent properties of a wildfire convective plume. J Appl Meteor Climatol.

[36] Galperin MY. Genome diversity of spore-forming firmicutes. Microbiol Spectr. 2013;1. 10.1128/microbiolspectrum.TBS-0015-2012. 10.1128/microbiolspectrum.TBS-0015-2012 PMC4306282 26184964

[37] Vaïtilingom M, Attard E, Gaiani N, Sancelme M, Deguillaume L, Flossmann AI (2012). Long-term features of cloud microbiology at the puy de Dôme (France). Atmos Environ.

[38] Favet J, Lapanje A, Giongo A, Kennedy S, Aung Y-Y, Cattaneo A (2013). Microbial hitchhikers on intercontinental dust: catching a lift in Chad. ISME J.

[39] Calderón-Ezquerro M, del C, Serrano-Silva N, Brunner-Mendoza C (2021). Aerobiological study of bacterial and fungal community composition in the atmosphere of Mexico City throughout an annual cycle. Environ Pollut.

[40] Bowers RM, Sullivan AP, Costello EK, Collett JL, Knight R, Fierer N (2011). Sources of bacteria in outdoor air across cities in the midwestern United States. Appl Environ Microbiol.

[41] Dworkin M, Falkow S, Rosenberg E, Schleifer K-H, Stackebrandt E, editors. The prokaryotes. New York, NY: Springer New York; 2006.

[42] Dove NC, Klingeman DM, Carrell AA, Cregger MA, Schadt CW. Fire alters plant microbiome assembly patterns: integrating the plant and soil microbial response to disturbance. New Phytol 2021;nph.17248. 10.1111/nph.17248 PMC8251558 33525047

[43] Wilkinson DM, Koumoutsaris S, Mitchell EAD, Bey I (2012). Modelling the effect of size on the aerial dispersal of microorganisms: modelling the aerial dispersal of microorganisms. J Biogeogr.

[44] Smith DJ, Griffin DW, McPeters RD, Ward PD, Schuerger AC (2011). Microbial survival in the stratosphere and implications for global dispersal. Aerobiologia.

[45] Boreson J, Dillner AM, Peccia J (2004). Correlating bioaerosol load with PM2.5 and PM10cf concentrations: a comparison between natural desert and urban-fringe aerosols. Atmosph Environ.

[46] Bhattarai H, Saikawa E, Wan X, Zhu H, Ram K, Gao S (2019). Levoglucosan as a tracer of biomass burning: recent progress and perspectives. Atmosph Res.

[47] Bowers RM, McCubbin IB, Hallar AG, Fierer N (2012). Seasonal variability in airborne bacterial communities at a high-elevation site. Atmosph Environ.

[48] Barberán A, Ladau J, Leff JW, Pollard KS, Menninger HL, Dunn RR (2015). Continental-scale distributions of dust-associated bacteria and fungi. Proc Natl Acad Sci USA.

[49] Frohlich-Nowoisky J, Pickersgill DA, Despres VR, Poschl U (2009). High diversity of fungi in air particulate matter. Proc Natl Acad Sci.

[50] Fröhlich-Nowoisky J, Burrows SM, Xie Z, Engling G, Solomon PA, Fraser MP (2012). Biogeography in the air: fungal diversity over land and oceans. Biogeosciences.

[51] Frohlich-Nowoisky J, Pickersgill DA, Despres VR, Poschl U (2009). High diversity of fungi in air particulate matter. Proceedings of the National Academy of Sciences.

[52] Vuono DC, Munakata-Marr J, Spear JR, Drewes JE (2016). Disturbance opens recruitment sites for bacterial colonization in activated sludge: disturbance opens recruitment for bacterial colonization. Environ Microbiol.

[53] Jones SE, McMahon KD (2009). Species-sorting may explain an apparent minimal effect of immigration on freshwater bacterial community dynamics. Environ Microbiol.

[54] Christner BC, Cai R, Morris CE, McCarter KS, Foreman CM, Skidmore ML (2008). Geographic, seasonal, and precipitation chemistry influence on the abundance and activity of biological ice nucleators in rain and snow. PNAS.

[55] Barry KR, Hill TCJ, Levin EJT, Twohy CH, Moore KA, Weller ZD (2021). Observations of ice nucleating particles in the free troposphere from Western US wildfires. J Geophys Res Atmosph.

[56] Joyce RE, Lavender H, Farrar J, Werth JT, Weber CF, D’Andrilli J, et al. Biological ice-nucleating particles deposited year-round in subtropical precipitation. Appl Environ Microbiol. 2019;85:e01567–19. 10.1128/AEM.01567-19 PMC6856338 31562166

[57] Kobziar LN, Thompson GR (2020). Wildfire smoke, a potential infectious agent. Science.

