# Supplementary material for: Wildland fire smoke alters the composition, diversity, and potential atmospheric function of microbial life in the aerobiome
Source: ISME Commun. 2022 Jan 25;2:8. doi: 10.1038/s43705-022-00089-5 (PMC9723787; doi:10.1038/s43705-022-00089-5)
Supplement: Supplementary file 1 — Supplemental Material [file 43705_2022_89_MOESM1_ESM.docx]

Supplemental Information - Appendix

**Wildland fire smoke alters the composition, diversity, and potential atmospheric function of microbial life in the aerobiome**

Leda N. Kobziar^1,a^, David Vuono^1,b^, Rachel Moore^c,d^, Brent Christner^d^, Timothy Dean^e^, Doris Betancourt^e^, Adam C. Watts^f^, Johanna Aurell^g^, Brian Gullett^e^

^a^Department of Natural Resources and Society, University of Idaho, Moscow, ID 83835;

^b^ Department of Civil and Environmental Engineering, Colorado School of Mines, Golden, CO 80401;

^C^Current Address: Earth and Atmospheric Sciences, Georgia Institute of Technology, Atlanta, GA 30332;

^d^Department of Microbiology and Cell Science, University of Florida, Gainesville, FL 32603;

^e^U. S. Environmental Protection Agency, Office of Research and Development, Research Triangle Park, NC 27711;

^f^Pacific Wildland Fire Sciences Laboratory, USDA Forest Service, Seattle WA 98103

^g^University of Dayton Research Institute, 300 College Park, Dayton, OH 45469

**SI Results**

Sequencing of 16S and 18S rRNA gene and fungal ITS regions generated an average of ~28,000 reads from a 20 L sample of smoke (S1). The average number of sequencing reads per sample in 16S rRNA libraries was significantly higher for smoke than ambient air (28,178 and 13,775, respectively; *t*-test; *P* < 0.05), but significant differences were not observed in the 18S rRNA (28,612 and 41,032, respectively; *t*-test; *P* = 0.052) or ITS (38,014 and 36,604, respectively; *t*-test; *p* = 0.55, respectively) libraries (Table S1). Despite the variability of sequencing read number across target regions, the total number of unique phylotypes recovered in the smoke samples were higher than those in ambient air: 959 vs 150 (16S), 296 vs. 90 (18S), and 93 vs. 31 (ITS), respectively (Fig. S1, Table S2). Of the phylotypes that were unique to each air type, the 16S smoke libraries had the largest fold change (5.4x) over libraries in ambient air. The higher number of 16S reads in smoke relative to ambient, but not in 18S or ITS libraries, and higher proportion of 16S phylotypes in smoke versus ambient, may have been confounded by the higher total cell number in smoke (previous section). However, overall 16S phylotype richness and evenness did not vary consistently with total cells numbers (Mantel: *r_M_* = -0.06, *p* = 0.72 [jaccard]; *r_M_* = -0.06, *p* = 0.72 [bray]) indicating that the higher cell numbers in smoke did not inflate phylotype richness and evenness. Archaea comprised 0.4% of total sequences in the 16S rRNA libraries and were only detected in smoke samples. The only other archaeal phylotype identified was *Halonotius*, a halophilic archaeon (Table S3) within the *Euryarchaeota*.

| **Table S1.** Phylotaxa found in Blanks that were removed from all analyses of Smoke and Ambient air | |
| --- | --- |
| 16S | Streptococcus; s__salivarius-thermophilus  Propionibacterium; s__acnes  Bacillus; s__cereus-pseudomycoides-toyonensis  Staphylococcus; s__capitis-caprae-epidermidis  Corynebacterium; s__pseudogenitalium-tuberculostearicum  Pelomonas; s__saccharophila  Afipia-Bradyrhizobium; s__NA  Kocuria; s__marina  Intrasporangiaceae; g__NA; s__NA  Acinetobacter; s__ursingii  Meiothermus; s__silvanus |
| 18S | (2) Tetrapoda;__Mammalia  Saccharomycetes; __Saccharomycetales  Brassicales; __Brassica  Malassezia;__uncultured fungus  (2) Nucletmycea;__Fungi  Ajellomycetaceae; __Ajellomyces  Chloroplastida; __Charophyta  SAR;_Alveolata  (11) Unassigned |
| ITS | (2) Cladosporium_grevilleae  Cladosporium_sp  Sarocladium_strictum  Sterigmatomyces_halophilus  Malassezia_japonica |

| **Table S2.** Summary statistics for sequence read and taxa by sample type (Ambient vs. Smoke) and by target region (mean ± s.d.) | | | | | | | | |
| --- | --- | --- | --- | --- | --- | --- | --- | --- |
|  | 16S | |  | 18S | |  | ITS | |
|  | Ambient | Smoke |  | Ambient | Smoke |  | Ambient | Smoke |
| Read statistics | 13,775 ± 11,990 | 28,178 ±  9,746 |  | 28,612 ± 16,186 | 37,058 ± 18,032 |  | 38,014 ± 23,536 | 36,604 ± 21,432 |
| Total reads | 110,203 | 479,020 |  | 228,892 | 926,448 |  | 304,109 | 622,276 |
| Total samples | n=8 | n=17 |  | n=8 | n=17 |  | n=8 | n=17 |
| Total phylotypes | 201 | 1010 |  | 123 | 329 |  | 40 | 102 |
| Unique phylotypes | 150 | 959 |  | 90 | 296 |  | 31 | 93 |
| Fold change (unique) | 5.4 | |  | 2.3 | |  | 2.0 | |

**Figure S1.** 16S Rarefaction curves for Ambient (left column) and Smoke (right column) with full sequencing depth (top row) and rarefied to sample with lowest sequencing depth, 860 reads (bottom row). Grey boxes in top plots show the approximate scale that is plotted in the bottom plots.

**Figure S2.** Phylum-level taxonomic resolution of Bacteria (A) and Family-level resolution of major phyla (B). Within-Family abundances are preserved at the phylum level.

**Figure S3.** PCoA split plot ordination of sample dispersion (left) and bacterial taxa dispersion (right) of Bray-Curtis dissimilarities. Alphaproteobacteria and Actinobacteria drive the majority of the separation between ambient and smoke libraries.

**Figure S4.** Abundance of the top 35 most abundant species-level ASVs, belonging to either Ambient or Smoke libraries, that are detected in at least 20% samples. Solid bars within boxplot represent the median read abundance and colored diamonds represent the mean read abundance of each sample type.

**Figure S5.** Phylogenetic tree of the top 16S Bacterial Families. Membership of families found in either ambient air (circles) or smoke (triangles) is shown and size of symbol corresponds to number of taxa.

**Figure S6.** Taxonomic resolution of 18S (A) and ITS (B). In A, within-Family abundances are preserved at the phylum level. In B, within-Order abundances of Ascomycota are preserved at the phylum level and within-Class abundances of Basidiomycota are preserved at the phylum level.
